# Improvement of anaerobic digestion of sewage mixed sludge using free nitrous acid and Fenton pre-treatment

**DOI:** 10.1186/s13068-018-1235-4

**Published:** 2018-08-28

**Authors:** Seyed Mostafa Hallaji, Ali Torabian, Behnoush Aminzadeh, Soraya Zahedi, Nicky Eshtiaghi

**Affiliations:** 1 School of Environment, College of Engineering, University of Tehran, Tehran, Iran; 2 Catalan Institute for Water Research (ICRA), Girona, Spain; 3 School of Engineering, Chemical and Environmental Engineering, RMIT University, Melbourne, Australia

**Keywords:** Anaerobic digestion, Free nitrous acid, Fenton reagent, Methane production, Sewage sludge

## Abstract

**Background:**

Recently, it has been indicated that free nitrous acid (FNA) and Fenton pre-treatment of waste activated sludge can enhance methane production in anaerobic digestion of waste activated sludge. In addition, it has been revealed that the substances used in these pre-treatments are both eco-friendly and economically attractive because not only are they produced in anaerobic digestion, but they are also low priced. Since primary sludge and waste activated sludge are mixed prior to anaerobic digestion in the majority of wastewater treatment plants, this study aims to assess the influence of combined FNA and Fenton on the anaerobic digestion of mixed sludge.

**Results:**

According to this study’s results, methane generation from anaerobic digestion of mixed sludge was enhanced when using FNA and Fenton pre-treatment, affirming the effectiveness of the individual and combined pre-treatments in anaerobic digestion of mixed sludge. The enhanced methane production was significant in combined pre-treatments (up to 72%), compared with FNA and Fenton pre-treatment alone (25% and 27%, respectively). This corroborates the positive synergistic effect of the combined pre-treatments on methane production. The enhanced methane can be attributed to augmented soluble fractions of organic matter in addition to increased readily biodegradable organic matter, caused by the pre-treatments. Additionally, the amount of chemical oxygen demand (COD) was assessed during anaerobic digestion, and it was revealed that COD decreased considerably when the pre-treatment strategies were combined.

**Conclusions:**

This study reveals that the pre-treatments are potentially applicable to full-scale wastewater treatment plants because a mixture of primary sludge and waste activated sludge was used for the pre-treatments. Additionally, combined FNA and Fenton pre-treatments prove more effective in enhancing methane production and organic removal than these pre-treatments alone. The enhanced methane production is important for two reasons: a higher amount of renewable energy could be generated from the enhanced methane production and the COD of digested sludge reduces in such a way that facilitates application of the sludge to agricultural lands and reduces sludge transport costs.

**Electronic supplementary material:**

The online version of this article (10.1186/s13068-018-1235-4) contains supplementary material, which is available to authorized users.

## Background

Today, wastewater treatment plants play a pivotal role in protecting water streams from pollution. Activated sludge process is a conventional method for treating wastewater, mainly because of its simplicity and high efficiency in removing organic matter from wastewater [1]. However, this process produces a great volume of sludge [2]. This is an important issue because managing the produced sludge entails allocating a great deal of money that sometimes can be as high as 50% of total operating costs in wastewater treatment plants [2, 3]. Furthermore, digestion of sludge, especially waste activated sludge, is often restricted by poor digestibility and slow fermentation process [4–6]. Consequently, adopting a pragmatic approach for addressing these issues in wastewater treatment plants is crucial.

As an influential method for dealing with sewage sludge, anaerobic digestion not only produces renewable energy from methane, but it also dispenses with aeration amenities and their related cost and energy. However, biochemical methane production of sewage sludge, particularly waste activated sludge, is limited due to lack of readily biodegradable organic matter for anaerobic organism consumption [4, 5]. To address this problem, diverse strategies have also been employed, such as chemical, mechanical, and enzymatic pre-treatment of sewage sludge prior to anaerobic digestion [7–10]. The intermediate-treatment and post-treatment of sewage sludge are also currently used strategies [11, 12]. These treatments disrupt cell walls and extracellular polymeric substance (EPS), accounting for around 70–80% of proteins and carbohydrates in sewage sludge. This provides an adequate amount of soluble organic matter for better performance of anaerobic organisms and methane production [2, 13–15]. Nevertheless, most of the mentioned methods are either eco-unfriendly or energy consuming [16].

Free nitrous acid (FNA) or HNO_2_ is an economically attractive, eco-friendly substance produced by nitration liquor anaerobic digestion [16]. FNA generates free radicals, such as peroxide (${\text{H}}_{2} {\text{O}}_{2}$), peroxynitrite (${\text{ONOO}}^{ - }$), nitrogen dioxide ($^{\cdot}{\text{NO}}_{2}$), hydroxide ion ($^{\cdot} {\text{OH }}^{ - }$), and nitric oxide ($^{\cdot} {\text{NO}}$) that disrupt cells and EPS, converting them to a soluble phase that is more readily biodegradable for anaerobic organisms [17, 18]. Zahedi et al. [9] indicated that 5-h exposure time to FNA pre-treatment could decrease cell viability by around 80% in waste activated sludge and enhance methane production in anaerobic digestion by 27%.

Fenton reaction, as an advanced oxidation process, is formed by H_2_O_2_ and Fe^+^ where Fe^+^ functions as a catalyst for producing strong free radicals like ${\text{OH}}^{ - } \cdot$ [19]. As far as oxidation–reduction potential is concerned, the free radicals produced by Fenton reagent (+ 2.33 V) are stronger than those produced by H_2_O_2_ alone (+ 1.36 V) and O_3_ (+ 2.07 V) [20]. Analogously, Fenton reagent disrupts cell walls and EPS, resulting in enhanced biodegradability of organic matter and methane production in anaerobic digestion of sewage sludge [21–23]. Hydrogen peroxide can also be produced by an electrochemical process in wastewater treatment plants [24]; this is of great importance because it paves the way for using Fenton pre-treatment in the anaerobic digestion process with lower costs. The Fenton reactions are demonstrated in Eqs. (1) and (2).

1 $${\text{Fe}}^{ 2+ } + {\text{ H}}_{ 2} {\text{O}}_{ 2} \to {\text{ Fe}}^{ 3+ } + {\text{ HO}}^{\cdot} + {\text{ OH}}^{ - }$$

2 $${\text{Fe}}^{ 3+ } + {\text{ H}}_{ 2} {\text{O}}_{ 2} \to {\text{ Fe}}^{ 2+ } + {\text{ HOO}} ^{\cdot} + {\text{ H}}^{ + }$$

This study investigated the influence of FNA and Fenton pre-treatments on anaerobic digestion of mixed sludge. Soluble fractions of organic matter in mixed waste activated sludge and primary sludge, before and after treatment, were measured to inspect the availability of readily biodegradable organic matter to anaerobic organisms. Furthermore, the amount of the biogas and methane production together with chemical oxygen demand (COD) were measured during anaerobic digestion to assess the effectiveness of the pre-treatments employed. To our knowledge, this is the first study evaluating the effectiveness of individual and combined FNA and Fenton pre-treatments on anaerobic digestion of mixed sludge.

## Results

### Sludge sources

The mixed sludge and inoculum used in this study were collected from the south wastewater treatment plant of Tehran. In this plant, primary sludge and waste activated sludge are mixed before anaerobic digestion with the ratio of 40:60 V/V. The same ratio for the mixed sludge was considered in this study.

The inoculum was collected from six mesophilic anaerobic digesters with a total capacity of 53,400 m^3^. Mixed sludge (primary sludge + waste activated sludge) was collected from the inlet pipe to the digesters. COD, Soluble chemical oxygen demand (SCOD), total solids (TS), total suspended solids (TSS), volatile solids (VS), and volatile suspended solids (VSS) of the inoculum and mixed sludge were measured as soon as they arrived at the university laboratory. Table 1 demonstrates the characterizations of the sludge used in this study (with standard errors achieved from triplicate measurements).

**Table 1:** Characterizations of the inoculum and mixed sludge used in this study

Characterization	COD (g/L)	SCOD (g/L)	TS (g/L)	TSS (g/L)	VS (g/L)	VSS (g/L)	pH
Inoculum	38.2 ± 0.1	3.25 ± 0.01	31.6 ± 0.3	26.8 ± 0.3	25.1 ± 0.2	21.8 ± 0.3	7.65 ± 0.01
Mixed sludge	48.5 ± 0.2	4.25 ± 0.02	39.5 ± 0.4	35.7 ± 0.4	31.5 ± 0.4	29.1 ± 0.2	6.32 ± 0.02

### Pre-treatment constituents

In this study, FNA pre-treatment, Fenton pre-treatment and combination of FNA and Fenton pre-treatment of mixed sludge were implemented prior to anaerobic digestion process. The designated concentrations of the substances used in these pre-treatments are shown in Table 2.

**Table 2:** Designated concentrations for pre-treatments

Pre-treatments	FNA (mg FNA/L)	H_2_O_2_ (mg H_2_O_2_/g VS)	Fe^+^ mg Fe^+^/mg H_2_O_2_	pH
control	0	0	0	6.32
FNA	2.5	0	0	5.5
Fenton 1	0	2.5	0.0067	3
Fenton 2	0	5	0.0067	3
FNA + Fenton 1	2.5	2.5	0.0067	5.5 and 3
FNA + Fenton 2	2.5	5	0.0067	5.5 and 3

### Solubilisation of organic matter

**Fig. 1:**
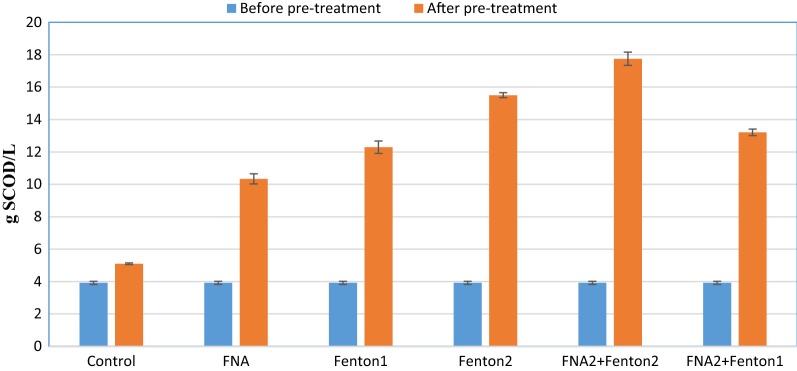
The amount of SCOD before and after pre-treatment. Error bars represent standard error achieved from triplicate tests

The influence of different pre-treatments on the substrate’s SCOD was assessed prior to biochemical methane potential tests. It was shown that the amount of SCOD increased dramatically in all pre-treated reactors (Fig. 1). The highest SCOD (17.75 g SCOD/L) was achieved in the BMP reactor treated by FNA + Fenton2. This was considerably higher than those obtained from FNA and Fenton pre-treated bioreactors alone. Comparing the effect of the individual pre-treatments, Fenton2 alone caused a considerably higher SCOD (15.5 g SCOD/L) than its FNA counterpart (10.34 g SCOD/L), suggesting a higher effectiveness of Fenton pre-treatment than FNA.

**Fig. 2:**
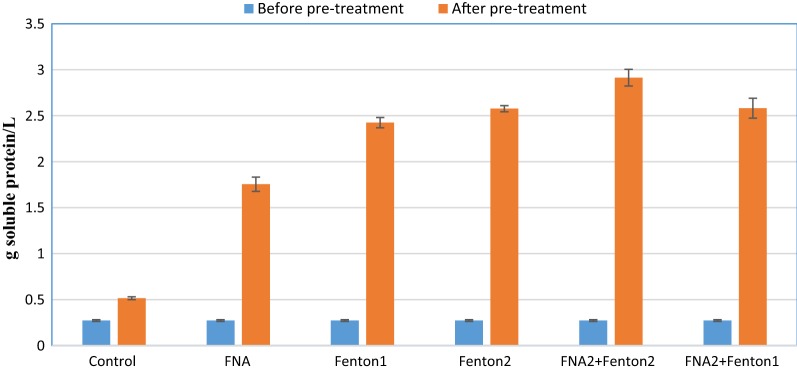
The amount of soluble protein before and pre-treatment. Error bars represent standard error achieved from triplicate tests

**Fig. 3:**
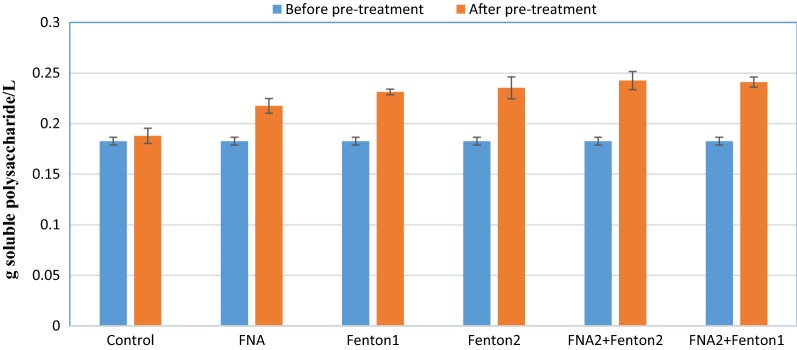
The amount of soluble polysaccharide before and after pre-treatment. Error bars represent standard error achieved from triplicate tests

Since not all types of organic matter are biodegradable for the anaerobic organisms, soluble fractions of protein and polysaccharide, the two important constituents of SCOD, were measured before and after treatments. Proteins and polysaccharides account for around 60% of the organisms’ constituents [19]. Thus, enhancement of soluble fractions of protein and polysaccharide after pre-treatments could affirm that more readily biodegradable organic matter is provided for anaerobic organisms due to disruption of cell walls and EPS in the substrate. The amounts of soluble protein and polysaccharide before and after treatments were measured in control and pre-treatment reactors (Figs. 2, 3). As the most significant augmentation, FNA + Fenton2 pre-treatment increased the amount of soluble proteins from 0.27 g protein/L to 2.9 g protein/L. The influence of Fenton2 and FNA + Fenton1 on solubalisation of proteins was identical that both increased soluble protein to 2.5 g protein/L. Most pre-treatments increased the soluble polysaccharide fraction by around 30%; however, the FNA pre-treatment increased the soluble polysaccharide fraction by 19%.

### Daily biogas production

**Fig. 4:**
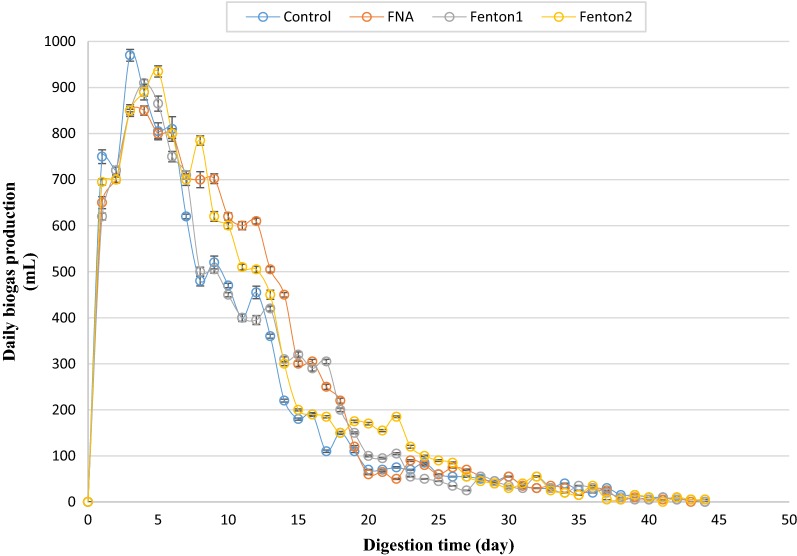
Daily biogas production from individual pre-treated bioreactors. Error bars represent standard error obtained from triplicate measurements

**Fig. 5:**
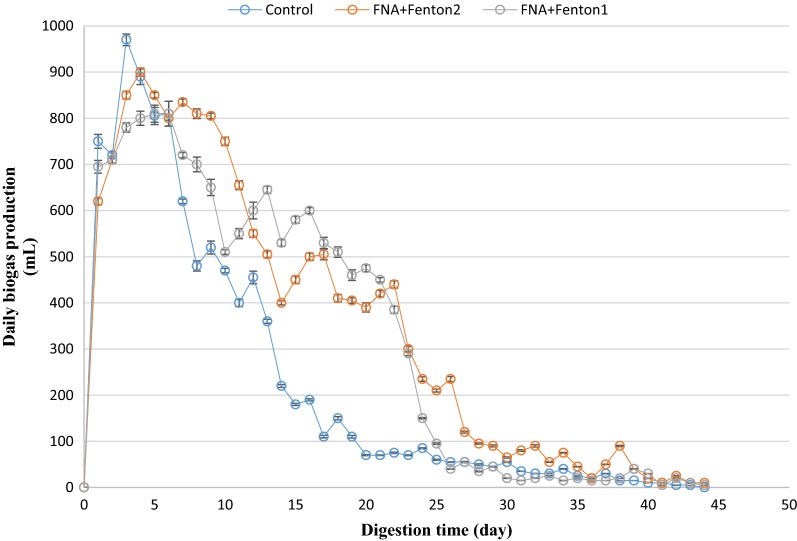
Daily biogas production from combined pre-treated bioreactors. Error bars represent standard error obtained from triplicate measurements

Daily biogas generation from different bioreactors was measured during the anaerobic digestion process. The total volume of biogas produced from the pretreated bioreactors was significantly enhanced in comparison to the control bioreactor (Figs. 4 and 5). However, during the first 5 days of the digestion process, the volume of biogas produced from the pre-treated rectors was slightly lower than that of the control, and it gradually overtook the volume of the control after 5 days. This is in agreement with previous studies in which a slight delay in biogas yield from pre-treated bioreactors was observed [18, 25, 26]. This is likely due to slight inhibitory effect on anaerobic organisms’ activity, which is produced by low nitrite/FNA concentration and probably also by overloading the pre-treated reactors with soluble organic matter that could produce a delay in the biogas production [27]. Therefore, although the maximum daily biogas production was obtained from the control bioreactor (970 mL in day 3), but the surface area below each chart, which represents the cumulative biogas production, was significantly higher in pre-treated reactors than that of the control at the end of the digestion. Accordingly, the FNA + Fenton2 pre-treated bioreactor achieved the highest volume of cumulative biogas production. This was also significantly higher than cumulative biogas from FNA and Fenton pre-treatment alone (*p* < 0.05).

### Cumulative methane production

**Fig. 6:**
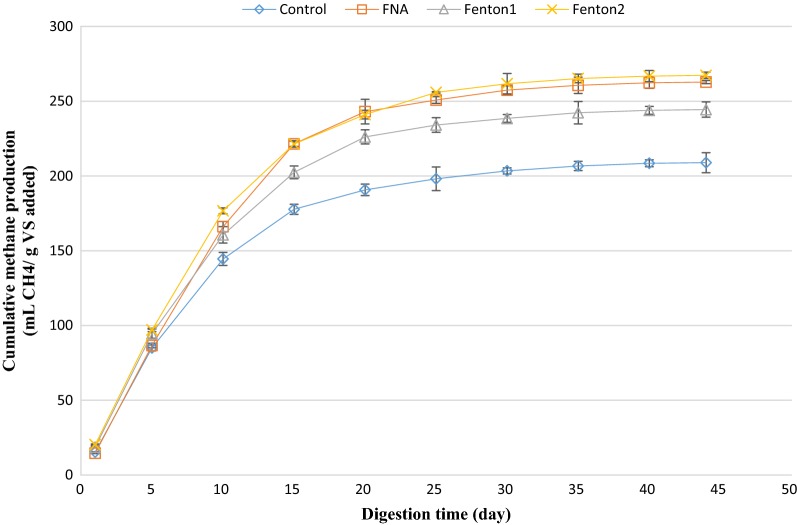
Cumulative methane production from singular pre-treated bioreactors. Error bars represent standard error obtained from triplicate measurements

**Fig. 7:**
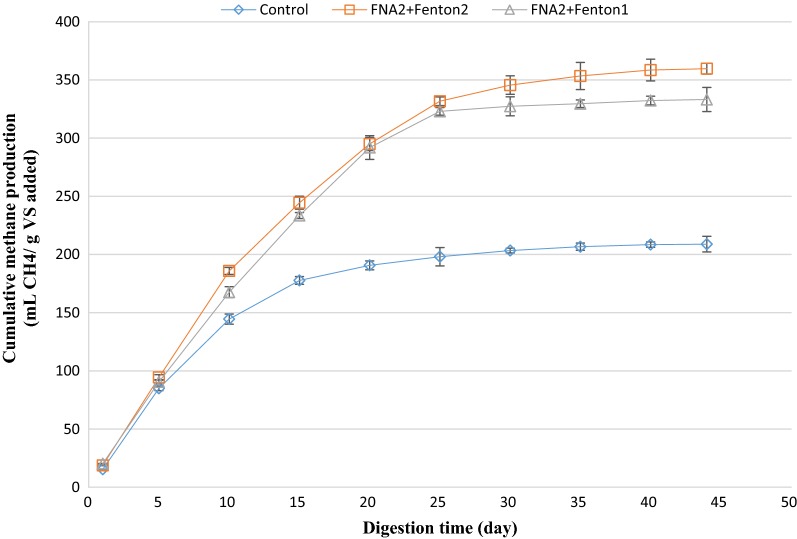
Cumulative methane production from combined pre-treated bioreactors. Error bars represent standard error obtained from triplicate measurements

The amount of cumulative methane production from anaerobic digestion of mixed sludge was regularly measured during the anaerobic digestion. Analogous to biogas production, the amount of methane production in the first 5 days of the digestion was slightly lower than that of the control reactor. However, the amount of methane production overtook that of the control gradually (Figs. 6 and 7). The considerably enhanced methane production from the pre-treated biochemical methane potential reactors could be attributed to the increased readily biodegradable organic matter that was available to anaerobic organisms due to disruption of cell walls. The highest cumulative methane production was obtained from combined FNA and Fenton pre-treated reactors. Accordingly, the amount of cumulative methane production from the biochemical methane potential reactor, pretreated with FNA + Fenton2 increased by 72% compared to the control. This was significantly higher than methane improvement obtained from these pre-treatments alone (*p* < 0.05), affirming the synergistic effect of combined FNA and Fenton pre-treatment. Followed by that, FNA + Fenton1 and Fenton2 pre-treatments caused the highest methane production, respectively, with 333 mL CH_4_/g vs. and 267 mL CH_4_/g vs. The methane/biogas yield fluctuated between 50 and 60% during the anaerobic digestion process, revealing that the enhanced cumulative methane production was mainly due to the enhanced biogas production and biodegradability of organic matter, not due to the enhanced methane content of the biogas. Analogously, the carbon dioxide/biogas yield did not experience a considerable change during the anaerobic digestion process (it fluctuated between 25 and 35%).

### COD removal efficiency

**Fig. 8:**
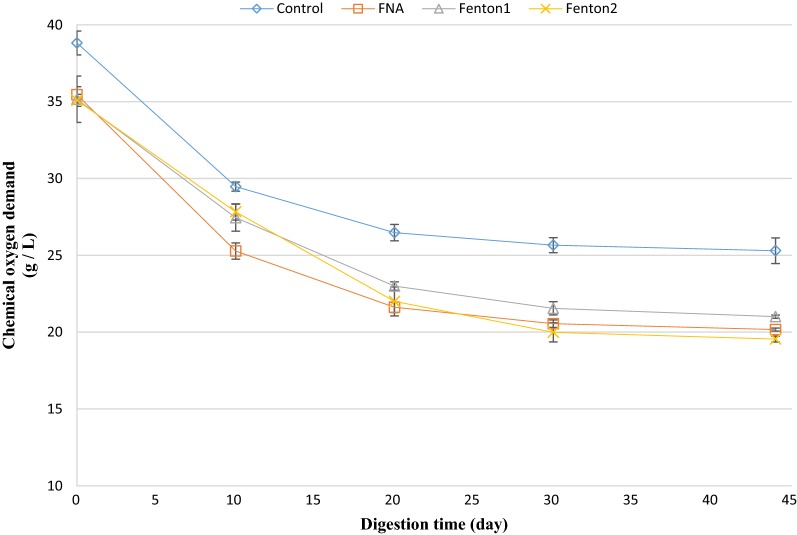
COD trend in singular pre-treated bioreactors. Error bars represent standard error obtained from triplicate measurements

**Fig. 9:**
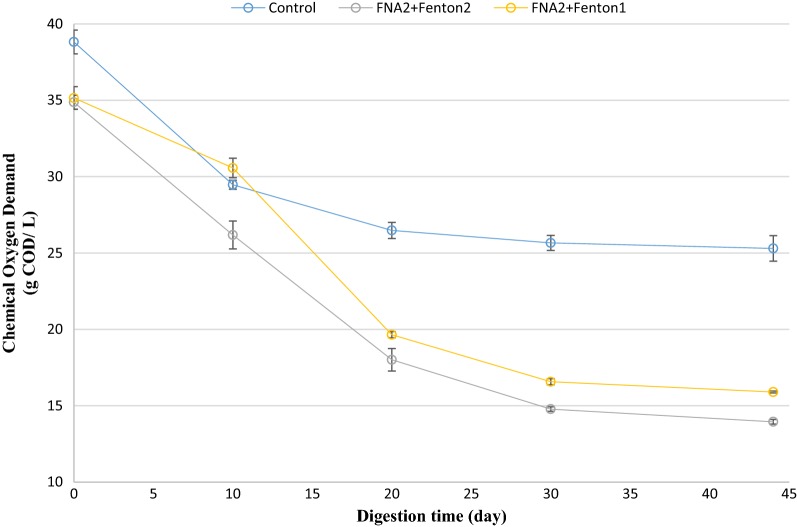
COD trend in combined pre-treated bioreactors. Error bars represent standard error obtained from triplicate measurements

The amount of COD in biochemical methane potential reactors was measured during the anaerobic digestion process. The amount of COD removal in the pre-treated reactors was considerably enhanced compared to the control reactor (Figs. 8 and 9). This could justify the higher methane and biogas production achieved in the pre-treated reactors. The highest COD removal efficiency was obtained by the FNA + Fenton2 pre-treated reactor, in which COD was significantly reduced by 59%, compared to the control (34% reduction) (*p* < 0.05). The other combined pre-treatment (FNA + Fenton1) caused the second highest COD removal of 54%. Furthermore, FNA pre-treatment was approximately as efficient as Fenton pre-treatment in removing COD with 43%, compared with 40% and 44% obtained by Fenton1 and Fenton2, respectively.

## Discussion

Having taken all aspects into account, the pre-treatments clearly increased the soluble fraction of organic matter. This stems mainly from oxidative properties of the pre-treatments employed in this study that disrupt cell walls and EPS [17, 19]. More organic matter was solubilized when higher concentrations of pre-treatment reagents were used. When it comes to effectiveness of the pre-treatments in solubilizing different types of organic matter, the pre-treatments were much more influential in solubilizing protein than polysaccharide. This can be attributed to biocidal effect of the pre-treatments that affect proteins more substantially than polysaccharide [9], as the main composition of organisms’ cell is around 50% protein [19]. The amount of soluble fraction of organic matter is enhanced slightly in the control reactor after pre-treatment time, suggesting some solubalisation with no chemical additives and pH control. The improvement of organic solubalisation is consistent with but slightly higher than the results achieved in previous studies, in which soluble fraction of organic matter was analogously augmented [9, 18, 25, 28–30]. This can be attributed to sludge characteristics and the concentration of the substances used in this study.

Whether alone or combined, FNA and Fenton pre-treatments enhanced methane production, affirming effectiveness of these pre-treatments in improving methane production from anaerobic digestion process. The methane enhancement was mainly because of augmented biogas production, not because of considerably enhanced methane content, due to the approximately constant methane/biogas yield. This stems from the release of more readily biodegradable organic matter in pre-treated bioreactors (Figs. 1, 2, 3). The higher concentrations of the pre-treatment constituents resulted in enhanced biogas production attributable to providing more soluble organic matter for anaerobic organisms. Combined pre-treatments achieved significantly higher methane production than FNA and Fenton pre-treatments alone (*p* < 0.05), indicating a positive synergistic effect of these pre-treatments on methane production. This could be because the free radicals released by each pre-treatment hydrolyze different types of organic matter in the substrate that resulted in enhanced biodegradability of organic matter and methane production in biochemical methane potential reactors. In all biochemical methane potential tests, a short period of lag time (5 days) was observed during which biogas production from pre-treated reactors gradually overtook that of the control. This could be attributed to low nitrite/FNA concentration and probably also to overloading the pre-treated reactors with soluble organic matter. From an economic and environmental perspective, the enhancement of methane production is of great significance because not only could higher renewable energy be generated, but also methane emission into the atmosphere as a major greenhouse gas could be decreased from anaerobic digestion of sewage sludge [31]. The amount of methane enhancement caused by individual FNA pre-treatment was 27% in Zahedi et al. [9] and 20% in Wang et al. [28]. In this study, a similar amount of methane enhancement was achieved (25%) from an individual FNA pre-treated bioreactor. The difference can be mainly attributed to the different digestion time, characterizations of the substrate and the inoculum employed in this study. The amount of methane enhancement caused by singular Fenton pre-treatment was 19.4% in Erden and Filibeli [23] and 15% in Pilli et al. [31]. A slightly higher amount of methane enhancement was achieved in this study with singular Fenton pre-treatment (27%), which further corroborate the effectiveness of Fenton pre-treatment in enhancing methane production from anaerobic digestion of the sewage sludge. Compared to combined FNA and thermal pre-treatment used in Wang et al. [28], the amount of maximum cumulative methane production was about 30% higher in this study. However, different sludge specifications and using higher dosages of FNA should be considered for more precise comparison.

The COD of digested sludge is one of the most important characteristics in applying the sludge to agricultural lands and forests. The amount of COD removal improved considerably in the biochemical methane potential reactors treated with FNA and Fenton. The improved COD is linked to higher biogas and methane production in the biochemical methane potential reactors because producing higher methane (in relatively constant methane/biogas yield) implies that higher amounts of organic matter have been consumed by anaerobic organisms, resulting in lower COD at the end of the digestion process. The enhanced COD removal in this study is of paramount importance because it lowers the amount of sludge and reduces the associated costs of transport. It also paves the way for shaping an integrated, sustainable system for treating sewage sludge by applying sludge to agricultural lands and forests.

Despite the significant results this research represents, applying new systems to anaerobic digestion of sewage sludge entails a great deal of money and time. Therefore, in future studies, a precise economic assessment for full-scale application of combined FNA and Fenton pre-treatments seems indispensable. Additionally, the influence of the pre-treatments on the microbial community should be put into perspective to provide deep insight into the mechanism of these pre-treatments and possible long-term side-effects on microbial behaviour.

## Conclusions

This study investigated the feasibility of enhancing anaerobic digestion of mixed primary sludge and waste activated sludge through combined FNA and Fenton pre-treatment. Combined FNA and Fenton pre-treatments were shown to increase soluble fractions of organic matter considerably more than these pre-treatments alone, resulting in enhanced biodegradability of organic matter, biogas production, methane production, and COD removal during the anaerobic digestion process. The improved methane production is of paramount importance, not only because higher amounts of renewable energy are obtained from the anaerobic digestion process, but also because lower methane emission, a major greenhouse gas, is released to the atmosphere. The improved COD of the digested sludge paves the way for having a more integrated and sustainable sludge treatment process, as sludge transport expenditures are reduced and the digested sludge achieves a higher potential application to agricultural lands. Additionally, combined FNA and Fenton pre-treatment is potentially an economically attractive and environmentally friendly technology, particularly considering that both are obtainable as by-products from anaerobic digestion of sewage sludge.

## Methods

### Analytical methods

COD, SCOD, VS, VSS, TS, and TSS were measured according to standard methods for the examination of water and wastewater [32]. Biopolymers (proteins and polysaccharides) were measured in the soluble phase before and after pre-treatment. To separate solids from liquids, sludge was centrifuged for 30 min at 10,000 rpm and the supernatant was filtrated through a 0.45 µm pore size glass fiber filter. Proteins were measured with the Folin Phenol Reagent according to Lowry [33] and Peterson [34]. Phenol with sulfuric acid was also used for measuring polysaccharide [35]. These methods have been widely used to determine the proteins and polysaccharides concentration, even in sludge pre-treatment studies to determine the effect of pre-treatments on sludge properties [9, 13, 25, 26, 36, 37].

The volume of biogas was measured by liquid displacement method [38]. The liquid barrier used in this method was 100% saturated with NaCl and acidified with H_2_SO_4_ (pH = 2) to reduce dissolution of the biogas (specially CO_2_ and CH_4_) in the liquid barrier and eliminate the errors in measuring the volume of biogas [38]. Gas chromatography (GC) with a thermal conductivity detector (TCD) at 100 °C and helium as a carrier gas was employed when analyzing the main biogas composition (CH_4_ and CO_2_). The biogas was collected from the bioreactors in Tedlar gas bags before using in GC measurements.

### FNA and Fenton methodology

One-liter reactors were used when carrying out FNA pre-treatment. FNA concentration used in this study was calculated by the equations $[{\text{FNA}}] = [{\text{NaNO}}_{2} ]/[{\text{Ka}} \times 10^{\text{pH}} ]$ and ${\text{Ka}} = \,e^{{ - 2300/(273\,\, + \,^\circ {\text{C}})}}$ [39]. In these equations, °C is the room temperature (≈ 25 °C), pH equals 5.5, and NaNO_2_ is the concentration of nitrite salt. The considered FNA concentration is obtained from economic assessments from literature review [18, 40–42]. With the FNA concentration, the nitrite salt concentration can be calculated. In this experiment, the pH of the mixed sludge was set at 5.5 ± 0.1 with 1 M HCL. Next, the calculated nitrite salt concentration was added to the treatment reactors, and the mixture was gently blended with a magnetic stirrer for 5 h [25].

Fenton pre-treatments were also conducted in 1-L reactors. The designated concentrations of H_2_O_2_ for the Fenton reaction were considered from the literature review [19, 23, 30, 31]. First, the pH of the mixed sludge was set to three by H_2_SO_4_ [22]. The designated Fe^+^ and H_2_O_2_ concentrations were then achieved by adding FeSO_4_ and H_2_O_2_ to the reactors, and the pre-treatment lasted for 1 h to obtain maximum solubalisation and biodegradability of organic matter, according to Pham et al. [43]. In the Fenton reaction, the ratio between H_2_O_2_ and Fe^2+^ was set at 0.0067, according to Erden and Filibeli [30] and Pham et al. [43].

For combined pre-treatments, the FNA pre-treatment was first conducted, and after 5-h exposure time, the Fenton pre-treatment with 1-h exposure time was conducted. To keep the pH of the reactors stable, the pH was regularly measured during the treatment process. During FNA pre-treatment, the pH of the mixture remained stable at 5.5, but the pH in the Fenton pre-treatment tended to fluctuate so that it was set at three again once it changed.

### Biochemical methane potential tests

**Fig. 10:**
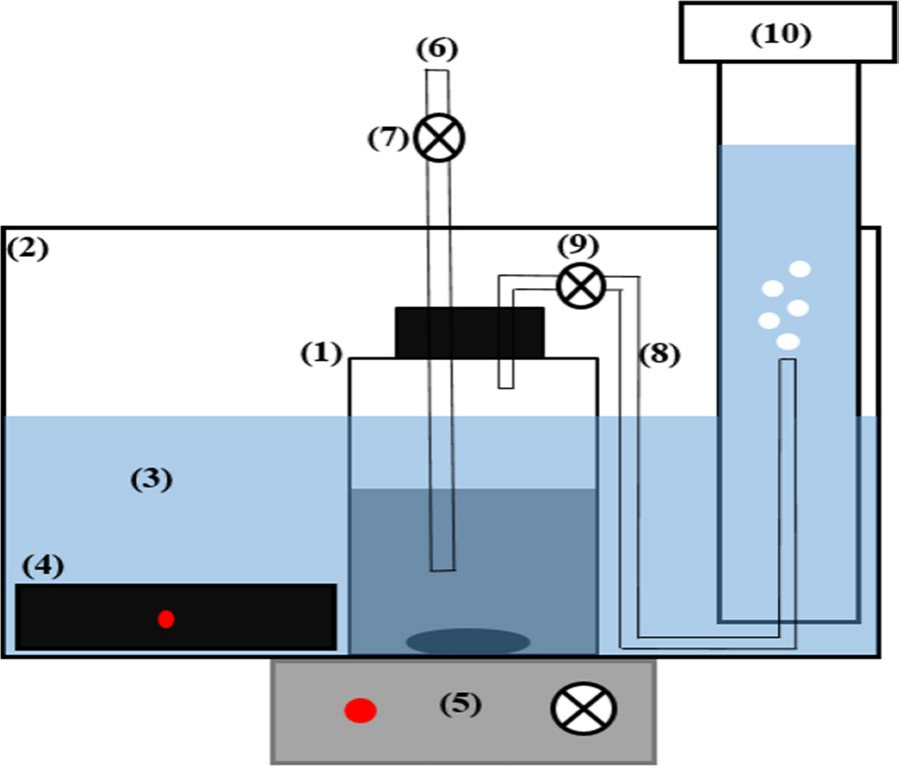
Schematic of experimental system. (1) Biochemical methane potential reactor, (2) aquarium, (3) saturated and acidified water, (4) automatic heater, (5) magnetic stirrer, (6) sampling pipe, (7) sampling control valve, (8) biogas collecting pipe, (9) biogas control valve, (10) graduated cylinder

For the biochemical methane potential tests, 18 bioreactors with 1000 mL capacity were considered with 500 mL working volume. Biochemical methane potential system is shown in Fig. 10. The ratio between inoculum and mixed sludge was 2 for proper performance of bioreactors, according to Boulanger et al. [44]. A control test (mixed sludge without chemical additives and pH control) was also conducted. Prior to mixing with the inoculum, the pH of the pre-treated mixed sludge increased to seven and maintained at 37 ± 1 °C to prevent pH and temperature shock to anaerobic organisms of the inoculum. After mixing the substrate and inoculum, the reactors were completely sealed to provide a strict anaerobic environment for better performance of anaerobic organisms. The reactors were put into a warm water bath heated by automatic heaters, and the temperature was set at 37 ± 1 °C. To provide uniform temperature and substrate distribution for better organism activity, the reactors were permanently mixed (100 rpm) by magnetic stirrers during the digestion process. The measurements were performed regularly for 44 days, when no biogas production was detected from the bioreactors (Additional file 1).

For assessing the influence of nitrite on inoculum performance, two sets of blanks were considered. Blank I, in which inoculum without chemical additives and substrate was used in biochemical methane potential tests. Blank II, identical to blank I except with the addition of nitrite stock solution, which resulted in an initial nitrite level of around 70 mg N–NO_2_–/L. Assessing bioreactors I and II, it was shown that the concentration of nitrite used in this study has no a significant effect on inoculum performance. This is in agreement with previous studies, in which no significant influences on inoculum performance were analogously observed [9, 18, 26, 28]. The methane production from mixed sludge was obtained by subtracting measured biogas production in an experimental bioreactor from that measured in blank I.

### Data analyses

To determine the significance of differences in the parameters studied, one-way factor Analysis of Variance ANOVA was used with significance levels of *p* < 0.05. Data analysis and graph processing were carried out with Microsoft Excel software (2010).

## Additional file

**Additional file 1.** Additional tables and figures.

## Abbreviations

COD: chemical oxygen demand
SCOD: soluble chemical oxygen demand
TS: total solid
TSS: total suspended solid
VS: volatile solid
VSS: volatile suspended solid
GC: gas chromatography
TCD: thermal conductivity detector
FNA: free nitrous acid
EPS: extra-cellular polymeric substance
