# Supplementary material for: Improvement of anaerobic digestion of sewage mixed sludge using free nitrous acid and Fenton pre-treatment
Source: Biotechnol Biofuels. 2018 Aug 28;11:233. doi: 10.1186/s13068-018-1235-4 (PMC6112153; doi:10.1186/s13068-018-1235-4)

**Supplementary data**

**Table 1-S. Soluble Chemical Oxygen Demand before and after pre-treatments (average of triplicate tests)**

| Reactors | Before PT (g/l) | After PT (g/l) |
| --- | --- | --- |
| Control | 3.92 | 5.1 |
| FNA | 3.92 | 10.34 |
| FEN1 | 3.92 | 12.29 |
| FEN2 | 3.92 | 15.5 |
| FNA+FEN2 | 3.92 | 17.75 |
| FNA+FEN1 | 3.92 | 13.21 |

**Table 2-S. Soluble protein before and after pre-treatments (average of triplicate tests)**

| Reactors | Before PT | After PT |
| --- | --- | --- |
| Control | 0.27154 | 0.51436 |
| FNA | 0.27154 | 1.75432 |
| FEN1 | 0.27154 | 2.42341 |
| FEN2 | 0.27154 | 2.57633 |
| FNA+FEN2 | 0.27154 | 2.91277 |
| FNA+FEN1 | 0.27154 | 2.58064 |

**Table 3-S. Soluble polysaccharide before and after pre-treatments (average of triplicate tests)**

| Reactors | Before PT | After PT |
| --- | --- | --- |
| Control | 0.18266 | 0.187902 |
| FNA | 0.18266 | 0.217566 |
| FEN1 | 0.18266 | 0.231257 |
| FEN2 | 0.18266 | 0.235389 |
| FNA+FEN2 | 0.18266 | 0.242607 |
| FNA+FEN1 | 0.18266 | 0.24101 |

**Table 4-S. Daily biogas production** **during digestion process (average of triplicate tests)**

| day | Control | FNA | FEN1 | FEN2 | FNA+FEN2 | FNA+FEN1 |
| --- | --- | --- | --- | --- | --- | --- |
| 0 | 0 | 0 | 0 | 0 | 0 | 0 |
| 1 | 750 | 650 | 620 | 695 | 620 | 695 |
| 2 | 720 | 700 | 720 | 700 | 710 | 710 |
| 3 | 970 | 850 | 850 | 850 | 850 | 780 |
| 4 | 890 | 850 | 910 | 890 | 900 | 800 |
| 5 | 805 | 800 | 865 | 935 | 850 | 810 |
| 6 | 810 | 800 | 750 | 800 | 800 | 810 |
| 7 | 620 | 710 | 710 | 700 | 835 | 720 |
| 8 | 480 | 700 | 500 | 785 | 810 | 700 |
| 9 | 520 | 702 | 505 | 620 | 805 | 650 |
| 10 | 470 | 620 | 450 | 600 | 750 | 510 |
| 11 | 400 | 600 | 400 | 510 | 655 | 550 |
| 12 | 455 | 610 | 395 | 505 | 550 | 600 |
| 13 | 360 | 505 | 420 | 450 | 505 | 645 |
| 14 | 220 | 450 | 310 | 300 | 400 | 530 |
| 15 | 180 | 300 | 320 | 200 | 450 | 580 |
| 16 | 190 | 305 | 290 | 190 | 500 | 600 |
| 17 | 110 | 250 | 305 | 185 | 505 | 530 |
| 18 | 150 | 220 | 200 | 150 | 410 | 510 |
| 19 | 110 | 120 | 150 | 175 | 405 | 460 |
| 20 | 70 | 60 | 100 | 170 | 390 | 475 |
| 21 | 70 | 65 | 95 | 155 | 420 | 450 |
| 22 | 75 | 50 | 105 | 185 | 440 | 385 |
| 23 | 70 | 90 | 55 | 120 | 300 | 290 |
| 24 | 85 | 80 | 50 | 100 | 235 | 150 |
| 25 | 60 | 60 | 45 | 90 | 210 | 95 |
| 26 | 55 | 75 | 35 | 85 | 235 | 40 |
| 27 | 55 | 70 | 25 | 55 | 120 | 55 |
| 28 | 50 | 55 | 55 | 45 | 95 | 35 |
| 29 | 45 | 40 | 45 | 40 | 90 | 45 |
| 30 | 55 | 55 | 35 | 30 | 65 | 20 |
| 31 | 35 | 35 | 30 | 40 | 80 | 15 |
| 32 | 30 | 30 | 55 | 55 | 90 | 20 |
| 33 | 30 | 35 | 30 | 25 | 55 | 25 |
| 34 | 40 | 30 | 20 | 20 | 75 | 15 |
| 35 | 25 | 15 | 35 | 15 | 45 | 20 |
| 36 | 20 | 30 | 30 | 35 | 20 | 15 |
| 37 | 30 | 25 | 20 | 5 | 50 | 15 |
| 38 | 15 | 5 | 10 | 5 | 90 | 20 |
| 39 | 15 | 10 | 5 | 15 | 40 | 40 |
| 40 | 10 | 5 | 5 | 10 | 20 | 30 |
| 41 | 10 | 5 | 10 | 0 | 10 | 5 |
| 42 | 5 | 10 | 5 | 10 | 25 | 20 |
| 43 | 5 | 0 | 5 | 5 | 10 | 10 |
| 44 | 0 | 5 | 0 | 5 | 10 | 5 |

**Table 5-S. Cumulative methane production** **during digestion process (average of triplicate tests)**

| Day | 1 | 5 | 10 | 15 | 20 | 25 | 30 | 35 | 40 | 44 |
| --- | --- | --- | --- | --- | --- | --- | --- | --- | --- | --- |
| Control | 15.4 | 85.0 | 144.5 | 177.7 | 190.7 | 198.1 | 203.4 | 206.7 | 208.5 | 208.9 |
| FNA | 14.6 | 86.6 | 166.1 | 221.5 | 243.0 | 250.8 | 257.4 | 260.6 | 262.3 | 262.8 |
| FEN1 | 18.6 | 94.5 | 160.6 | 202.4 | 226.1 | 234.1 | 238.5 | 242.3 | 243.9 | 244.4 |
| FEN2 | 20.3 | 97.0 | 176.7 | 221.4 | 241.1 | 255.9 | 261.7 | 265.2 | 266.8 | 267.3 |
| FNA+FEN2 | 18.8 | 94.4 | 185.9 | 244.5 | 295.0 | 331.7 | 345.6 | 353.4 | 358.5 | 359.7 |
| FNA+FEN1 | 20.3 | 90.6 | 167.6 | 233.5 | 291.9 | 323.0 | 327.4 | 329.6 | 332.3 | 333.2 |

| Day | 0 | 10 | 20 | 30 | 44 |
| --- | --- | --- | --- | --- | --- |
| Control | 38.82 | 29.47 | 26.48 | 25.66 | 25.30 |
| FNA | 35.45 | 25.28 | 21.62 | 20.55 | 20.17 |
| FEN1 | 35.16 | 27.45 | 23.00 | 21.55 | 21.01 |
| FEN2 | 35.09 | 27.83 | 22.00 | 19.99 | 19.55 |
| FNA+FEN2 | 34.87 | 26.18 | 18.01 | 14.78 | 13.95 |
| FNA+FEN1 | 35.15 | 30.57 | 19.65 | 16.57 | 15.90 |

**Table 6-S. Chemical Oxygen Demand during digestion process (average of triplicate tests)**

**GC samples:**


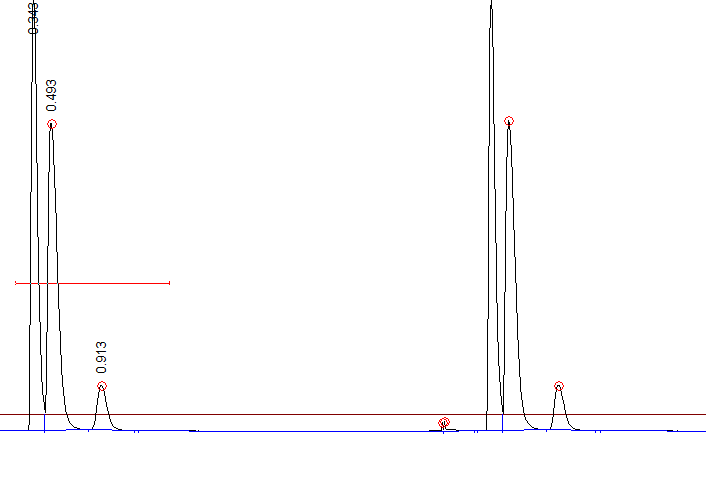


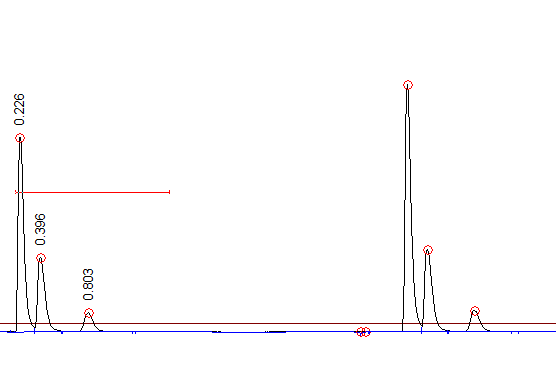


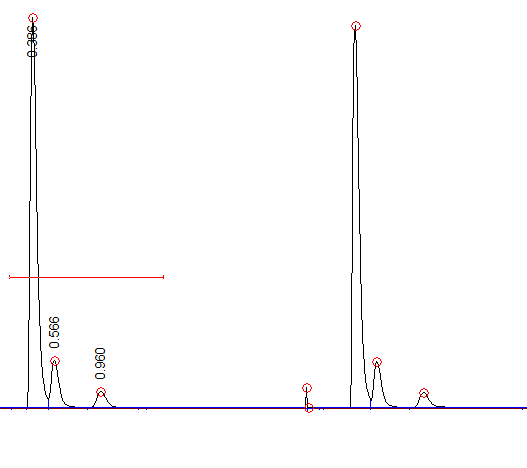

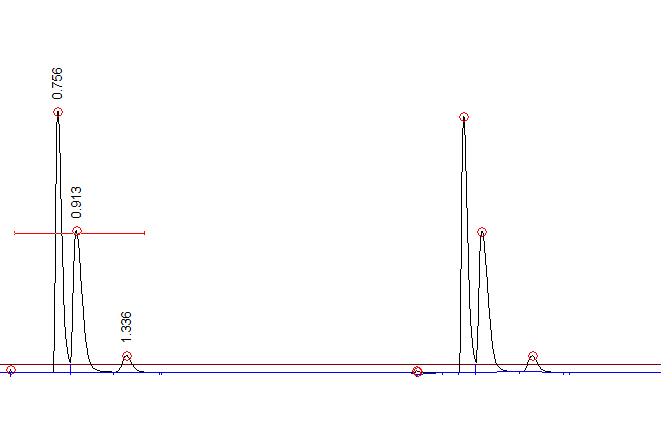


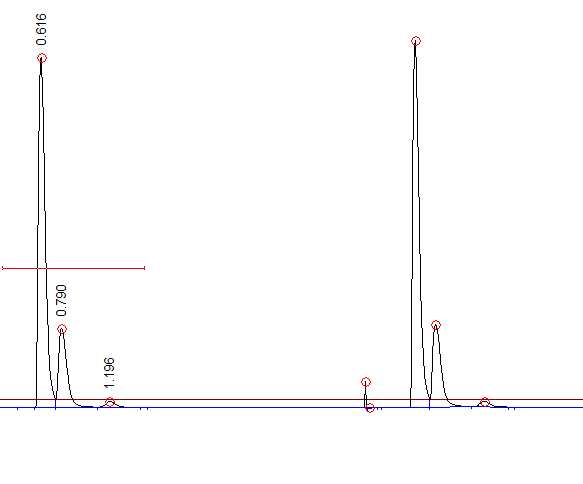

Supplement: Supplementary file 1 — Additional file 1. Additional tables and figures. [file 13068_2018_1235_MOESM1_ESM.docx]
